# Mobile Phones, Brain Tumors, and the Interphone Study: Where Are We Now?

**DOI:** 10.1289/ehp.1103693

**Published:** 2011-07-01

**Authors:** Anthony J. Swerdlow, Maria Feychting, Adele C. Green, Leeka Kheifets, David A. Savitz, International Commission for Non-Ionizing Radiation Protection Standing Committee on Epidemiology

**Affiliations:** 1 Section of Epidemiology, Institute of Cancer Research, Sutton, United Kingdom; 2 Karolinska Institutet, Institute of Environmental Medicine, Stockholm, Sweden; 3 Cancer and Population Studies Unit, Queensland Institute of Medical Research, Brisbane, Australia; 4 School of Translational Medicine, University of Manchester, Manchester, United Kingdom; 5 Department of Epidemiology, University of California at Los Angeles, Los Angeles, California, USA; 6 Department of Community Health, and; 7 Department of Obstetrics and Gynecology, Brown University, Providence, Rhode Island, USA

**Keywords:** brain cancer, cancer and radiation, epidemiology

## Abstract

Background: In the past 15 years, mobile telephone use has evolved from an uncommon activity to one with > 4.6 billion subscriptions worldwide. However, there is public concern about the possibility that mobile phones might cause cancer, especially brain tumors.

Objectives: We reviewed the evidence on whether mobile phone use raises the risk of the main types of brain tumor—glioma and meningioma—with a particular focus on the recent publication of the largest epidemiologic study yet: the 13-country Interphone Study.

Discussion: Methodological deficits limit the conclusions that can be drawn from the Interphone study, but its results, along with those from other epidemiologic, biological, and animal studies and brain tumor incidence trends, suggest that within about 10–15 years after first use of mobile phones there is unlikely to be a material increase in the risk of brain tumors in adults. Data for childhood tumors and for periods beyond 15 years are currently lacking.

Conclusions: Although there remains some uncertainty, the trend in the accumulating evidence is increasingly against the hypothesis that mobile phone use can cause brain tumors in adults.

In just 15 years the mobile phone has evolved from an uncommon, expensive, brick-shaped object used in restricted areas of Western countries to a convenient and ubiquitous part of modern life, with > 4.6 billion subscriptions worldwide (International Telecommunication Union 2010). The arrival of this mass technology has been accompanied by some public and media concern about the possibility that the radiofrequency (RF) fields emitted by the phones might cause cancer, especially brain tumors. Numerous committees have considered the evidence and recommended more research (Independent Expert Group on Mobile Phones 2000; Scientific Committee on Emerging and Newly Identified Health Risks 2009). Since 1999, a series of epidemiologic studies of mobile phone use and cancer have been published, mainly focused on brain tumor risks. Collectively, they have not provided evidence of a relationship, but they have had sufficient limitations to leave the question unresolved (Ahlbom et al. 2009).

The Interphone study was launched in 2000 to provide a more powerful and methodologically rigorous investigation of this issue by collecting data in 13 countries. Now, 10 years and ¤19 million later, after much anticipation and a lengthy delay, the key results on brain tumors have been published (Interphone Study Group 2010). What should be made of them, considered along with the rest of the literature? Do we now know whether mobile phones cause brain tumors? If not, how much closer are we to knowing?

## The Interphone Study

The Interphone study was an international, coordinated interview case–control study investigating the potential effect of mobile phone use on the risk of the two most common types of brain tumor, glioma and meningioma (and, although not yet published, also acoustic neuromas and parotid gland tumors). The study used a common core questionnaire and to some extent a common core protocol, but deviations and additions were allowed; for instance, cases were population based in most countries but hospital based in Japan and France, and controls were pair matched at nine centers but stratum matched in the other seven. These methodological inconsistencies add to the difficulty of interpreting the overall results. Nevertheless, the multicenter structure enabled a study of exceptional size: > 5,000 patients with these relatively uncommon tumors were interviewed in a 5-year period—a considerable feat.

The study questionnaire asked in detail about the type and pattern of use of each mobile phone the respondent had used and about other RF exposures and brain tumor risk factors. The questionnaire was administered by an interviewer using a computerized laptop data entry system (except in Finland), with practical advantages but with the disadvantage that no original paper records were available to check the fidelity of data entry for apparently erroneous values. The questionnaire collected information on hands-free phone use, which was excluded from analyses because head exposure would then be negligible. It is unknown, however, how well subjects can recall past use of hands-free devices or whether recall differed between cases and controls.

The analyses employed post hoc matching of one control per case (two for Germany) for the centers that had used a stratified control selection. The individual matching was then used for the analyses. This resulted in loss of data: 70 cases and > 2,000 interviewed controls were not included in the final analyses. Furthermore, most of the national studies that contributed to Interphone covered a wider age range (as low as 18 and/or up to 69 years) than the Interphone analyses (30–59 years), so a considerable proportion of the national data [e.g., 58% for Sweden (Lönn et al. 2005)] was not included in the overall pooled analyses. The national publications need to be considered, therefore, as additional semi-independent sources of evidence, not simply as subsets of the overall Interphone analysis.

The Interphone publication (Interphone Study Group 2010) compared 2,708 glioma cases diagnosed at 30–59 years of age during 2000–2004 with 2,972 controls, and 2,409 meningioma cases with 2,662 controls. Participation rates were 64% for glioma cases, 78% for meningioma cases, and 53% for controls, with considerable variation among study centers; proxies were used for 13% of glioma cases, 2% of meningioma cases, and 1% of controls. Sensitivity analyses did not suggest, however, that the results depended on participation rates across centers or on inclusion of proxies.

Key findings were a significantly diminished risk of both glioma and meningioma in regular users compared with people who were not users or were occasional users (“nonusers”); no trend in risk of either tumor type with cumulative hours of use but an apparent raised risk of glioma, and to a lesser extent meningioma, in those in the top decile of cumulative hours of use; and no relation of risk of either tumor type to cumulative number of calls, years of use, or years since first use. These results raise several important issues.

## Reduced Risk of Brain Tumors in Mobile Phone Users

The Interphone Study, as well as some previous case–control studies (Inskip et al. 2001; Muscat et al. 2000) and the only large cohort study (Schüz et al. 2006), identified a reduced risk of brain tumors among mobile phone users compared with nonusers. In the Interphone study as a whole, ever-regular use was associated with an odds ratio (OR) of 0.79 [95% confidence interval (CI): 0.68, 0.91] for meningioma and 0.81 (95% CI: 0.70, 0.94) for glioma. The pattern was consistent across the Interphone study sites and statistically precise, calling for explanation.

There is empirical evidence that the reduced risks were attributable partly to nonresponse bias (Vrijheid et al. 2009). Cases and controls who initially declined to participate but agreed to complete a short nonresponse questionnaire had lower frequencies of regular mobile phone use than did those who participated fully. The quantitative results from this nonresponse questionnaire imply that selection bias would produce an OR of 0.87–0.92 if the null hypothesis were true. It seems unlikely that differential response based on mobile phone use could explain the diminished risk entirely, because the reduction in risk was similar for study centers that did and did not reveal to potential participants the study’s focus on mobile phone use.

Even if the same pattern of diminished response by nonusers occurred for cases and controls, which it did not, the overall greater nonparticipation among controls because of refusal would result in a downward bias in the OR. Whereas only 11% of glioma and meningioma cases refused to participate, 30% of controls did so. Furthermore, the phone use of those who did not complete even the nonresponse questionnaire (e.g., because of refusal or death) is unknown, adding further uncertainty to the extent of the overall bias.

Other likely contributors to the diminished ORs in users are prodromal symptoms such as headaches and impaired cognition, which may have prevented recent initiation of mobile phone use among subjects with as yet undiagnosed brain tumors. Thus, some cases who would otherwise have become short-term users may have remained nonusers, leading to artifactually reduced ORs for brain tumor in phone users, especially short-term users (and low cumulative users, because short-term use will tend to result in low cumulative use). It seems likely that this accounts for at least part of the decreased risk in users, because the strongest reduction in glioma risk was found in the shortest-term users. Other potential contributors to diminished ORs can be hypothesized, but there is no evidence for them [see Supplemental Material, p. 1 (http://dx.doi.org/10.1289/ehp.1103693)].

The appropriate analytic approach and interpretation in light of this presumably noncausal reduction in risk are not obvious. One suggested response has been to alter the referent group, by using low regular use rather than nonuse plus occasional use as the referent. This results in an upward shift in the ORs across the board, more for glioma than for meningioma, but no change in the magnitude of those ORs relative to one another across the range of exposure (Interphone Study Group 2010). However, whether this decreases or increases the bias depends on two factors: whether the diminished risk is attributable to nonresponse, and whether the biases apply to low-level users as well as nonusers. Neither of these factors is known, but to the extent that the diminished risk is attributable to prodromal symptoms, changing the referent group would produce upward bias. If short-term users (or low cumulative users) are used as the referent exposure group, the more pronounced risk reduction in this group caused by prodromal symptoms would bias relative risks for long-term users (or high cumulative users) upward.

## Risks after Prolonged and Heavy Mobile Phone Use

If exposure to RF fields through mobile phone use were tumorigenic, people using mobile phones longest and those who were the heaviest users would be expected to show the highest risks of brain tumors. Reliability of recall of amount of use a decade ago is unknown, and the average amount of use is likely to have shifted over time as phone use has escalated universally. Validation studies of recall of phone use in the previous 6 months, and up to approximately 5 years in the past, have found that even in the short term, subjects on average underestimate the number of calls per month but overestimate duration of calls, with moderate systematic error (underestimation by light users, overestimation by heavy users) and a large amount of random error (Vrijheid et al. 2006). Recall of number of calls was found to be better than recall of their duration. Furthermore, in the Interphone study cases more often than controls gave implausibly high estimates of daily time spent on calls (e.g., 10 cases and no controls reported average use of > 12 hr/day). A validation study that included both cases and controls found that cases overestimated phone use in distant time periods, which could cause positive bias in risk estimates (Vrijheid et al. 2009). It thus appears that recall of amount of use was appreciably erroneous and quite likely different for cases than for controls. It is possible that recall of year of first use, and hence duration of use, may have been more reliable than recall of amount of use.

Notwithstanding the inherent unreliability of recalled amount of use, the only cumulative mobile phone exposure measures available in the Interphone study were duration and amount. Neither yielded material evidence of a positive association with brain tumors. Specifically, for the longest-term users (≥ 10 years since first use), no association was found for glioma (OR = 0.98; 95% CI: 0.76, 1.26) or meningioma (OR = 0.83; 95% CI: 0.61, 1.14). Most ORs were < 1.0, and no dose–response pattern was found. This is consistent with results from a cohort study based on subscriber lists (Schüz et al. 2006) but in contrast with the raised risks for long-term use reported by Hardell et al. (2006a, 2006b). For heavy use measured by estimated total number of calls, again, there was no positive association with brain tumors: ORs were < 1.0 in all categories of number of calls, including those in the top decile, for both glioma and meningioma. For heavy use assessed by cumulative duration of calls, again, there was no dose–response effect for either type of tumor. For glioma, although the risk estimate for subjects in the highest decile of total call time (≥ 1,640 hr) was modestly raised at OR = 1.40 (95% CI: 1.03, 1.89), it was disjointed from the risk in the next heaviest users, the second highest decile, which had one of the lowest risk estimates: OR = 0.71 (95% CI: 0.53, 0.96). Similarly, for meningioma the OR in the highest decile of total call-time OR was 1.15 (95% CI: 0.81, 1.62), whereas in the next heaviest decile of users it was 0.76 (95% CI: 0.54, 1.08). Furthermore, the top decile category presented was not actually 10% of the control data—it is unknown to what extent risk would have been raised in the true top decile, or to what extent the raised risk is a function of the cut-point chosen (about the 7th percentile for meningioma and the 8th percentile for glioma).

The only previously available risk estimates among comparably heavy users are from case–control studies conducted by Hardell et al. (2006a, 2006b) in Sweden, which reported a markedly raised risk and positive dose–response gradient for “malignant tumors” but not for meningioma. We have discussed elsewhere why the Hardell et al. results are problematic (Ahlbom et al. 2009). Assessment of the findings with respect to cumulative call time in individual published component studies of Interphone, whose participants variously covered a wider range of ages than Interphone, confirmed the lack of dose– response effect with glioma [see Supplemental Material, p. 2 (http://dx.doi.org/10.1289/ehp.1103693)]. Furthermore, for number of calls, which validation studies suggest may be better reported than cumulative hours of exposure, there was no indication of raised risk in the top decile or of dose response.

Finally, participants who had been using mobile phones the longest (≥ 10 years) and had accumulated highest lifetime call hours (≥ 1,640 hr) might be expected *a priori* to have been at the highest risk if RF exposure were tumorigenic. This was not the case, however, for either glioma (OR = 1.34; 95% CI: 0.90, 2.01) or meningioma (OR = 0.95; 95% CI: 0.56, 1.63) (Interphone Study Group 2010). Instead, it appeared that the very few individuals who started regular use only 1–4 years ago yet whose cumulative call time fell in the highest decile, because of their reported recent heavy use, carried the greatest risk of both tumor types: for glioma OR = 3.77 (95% CI: 1.25, 11.4) and for meningioma OR = 4.80 (95% CI: 1.49, 15.4), with no dose response. The similarity of the results for meningioma and glioma suggests that shared recall bias exists, because such a recent use period should have little or no bearing on the pathogenesis of meningioma, which tends to have a long latent period.

The magnitudes of relative risk of glioma and meningioma found in the top decile of cumulative use of phones were not large (1.40 and 1.15, respectively) and are on the margins of what epidemiology can detect. It is at a level at which the errors and biases identified in the study data provide a plausible—indeed, at present a more plausible—alternative explanation of the findings than does causation. Furthermore, the analyses were derived from a very large number of comparisons investigated [some reported by the Interphone Study Group (2010), the great majority not]; hence, there was the potential for selective emphasis in presentation of the results.

In summary, the Interphone study and the literature overall have methodological deficiencies but do not demonstrate greater risk of either glioma or meningioma with longer or greater use of mobile phones, although the longest period since first use examined is < 15 years.

## Anatomic Distribution of the Tumors Compared with Anatomic Distribution of Exposure

RF exposure during mobile phone use is highly attenuated within a few centimeters in the brain, so exposure is largely to the side of the brain, and to the anatomic area, closest to the antenna. It has been reported that on the side of the brain where the phone is used, 50–60% of the total RF energy is absorbed in the temporal lobe, and the average specific absorption rate is highest in the temporal lobe and the cerebellum (Cardis et al. 2008). Thus, examination of location of the tumor in relation to location of exposure is of interest.

*Laterality.* If there were a causal association between mobile phone use and brain tumor risk, one would expect an increased risk on the same side of the head as the phone is held and a null finding on the opposite side. On the other hand, if some brain tumor patients believed that mobile phone use had caused their tumor, and consequently overreported use on the affected side, this would result in an apparent risk increase on the same side of the head accompanied by a decreased risk on the opposite side. (The same bias is not possible for controls, who do not have a tumor side.) Furthermore, if there were a causal relationship, one would expect an effect of laterality to occur after a sufficient induction period, not for solely recent use (unless there was a promotional effect of mobile phones that was very rapid and substantial, which presumably would be easily and rapidly detectable from population incidence trends).

ORs for glioma and meningioma in the Interphone study tended to be greater in subjects who reported usual phone use on the same side of the head as their tumor than on the opposite side for most categories of duration of use, cumulative call time, and cumulative number of calls. Most ipsilateral ORs were not above unity, however, and there was no dose–response trend, although the greatest ORs tended to be for the top decile of ipsilateral exposure.

There are currently no validation studies of retrospective self-reported side of use, and there is no evidence of consistency over time in the preferred side of use. Overall, the greater risk for reported ipsilateral than contralateral use would be compatible with causation or bias as an explanation, but the finding that contralateral risks and many of the ipsilateral risks were generally below unity, with no consistent pattern of greater ipsilateral/contralateral ratios with greater exposure (except for cumulative number of calls and risk of glioma), would favor bias as the explanation.

*Lobe.* The risk of glioma in the temporal lobe for regular use and for most categories of exposure was reduced and did not differ from that in other lobes. ORs for long-term use and highest cumulative call time, however, were somewhat greater in the temporal lobe than in other lobes. This is the pattern one would expect if there were a causal effect, although there was no suggestion of a dose–response effect for temporal tumors, which would also be expected if there were causality. No coherent pattern was observed for meningioma, for which the OR for temporal lobe tumors for regular use was somewhat lower than for other lobes, and there was no evidence of greater risk in the temporal than other lobes in other categories of use.

*Exact anatomic location of the tumor.* Interphone collected neuroradiologic information on the exact locations of brain tumors in the study. Although this has not been published for the study overall, it has been published for glioma for many of the study centers and for meningioma for one center. These analyses gave no indication of an association of tumor risk to proximity of the tumor to the exposure source (Larjavaara et al. 2011; Takebayashi et al. 2008).

In summary, among the three types of data on anatomic location, the results for laterality of phone use are the least interpretable. They are compatible with bias, or at least partly with causation, but do not give firm evidence for either. The evidence on lobe of glioma, but not of meningioma, is inconsistently in the direction that would be expected with causality, but not decisively so. The evidence on exact location of the tumor, which one would expect to give the most rigorous analysis because it has greater precision without bias, does not support a causal association.

Data on tumor risk in relation to type of mobile phone, and hence of exposure, have not suggested a relation [see Supplemental Material, p. 2 (http://dx.doi.org/10.1289/ehp.1103693)].

## Other Relevant Evidence

The biological literature on RF and cancer does not support an etiologic effect—extensive research has not established any biological mechanism by which RF fields, which are not mutagenic, could cause cancer, and animal experiments have given no replicable evidence for cancer causation in animals (Scientific Committee on Emerging and Newly Identified Health Risks 2009).

The major biases and uncertainties in interpretation of the Interphone study are similar to those in other interview-based case–control studies of brain tumors and mobile phones. The exceptional size of the Interphone study has not proved to be a critical strength—issues of bias and misclassification have proved far more important than tightness of CIs. Therefore, more studies of the same basic design as Interphone, based on recall of phone use, no matter how carefully designed and conducted, are unlikely to add materially to our knowledge. There are other epidemiologic designs that do not share these weaknesses (although they have others), whose results need consideration in relation to the uncertainties remaining after Interphone: studies of the effects of occupational and residential RF exposures, record linkage-based case–control and cohort studies of phone use, and trend analyses of brain tumor incidence rates in the general population.

The occupational studies, and those of cancer risk in relation to residential proximity to RF broadcasting towers, have not indicated any cancer risk, although they have been methodologically weak (Ahlbom et al. 2004).

Studies that have linked private noncorporate telephone subscription records to cancer registry records (in certain Nordic countries) (Auvinen et al. 2002; Schüz et al. 2006) or death records (in the United States) (Dreyer et al. 1999) have the strengths that they avoid recall bias and misclassification and avoid participation bias. They have the weaknesses, however, not present in interview case–control studies such as Interphone, that the subscription data exclude corporate subscriptions, which in the early years were likely often to have been held by heavy users, and that the named subscriber is not necessarily the user. These problems are likely to have diluted any true association. A U.S. cohort study (Dreyer et al. 1999) was halted 1 year after recruitment and so was essentially uninformative. A national records-based case–control study in Finland (Auvinen et al. 2002) based on very short durations of use found a borderline significantly raised risk of glioma in ever-users, with some evidence for a relation to analog not digital phone use. A Danish cohort study (Schüz et al. 2006) followed 420,000 phone subscribers over a period of 7–21 years and gave no indication of raised risk of glioma or meningioma or any trend in risk with duration since first use.

*Trend data.* Analyses of secular trends in brain tumor incidence, in countries that have had good-quality diagnostic facilities and cancer registration, can give powerful evidence constraining what can reasonably be proposed as an etiologic relationship. The dramatic rise in mobile phone use over a relatively short period of time provides an unusual opportunity to assess the potential for a causal effect on cancer occurrence through high-quality, unbiased descriptive epidemiologic data. Because substantial misclassification is inevitable in recall-based exposure information from the Interphone interviews, it follows that if the raised relative risk observed in the top decile of users in the Interphone study were caused by phone use, not by chance or artifact, then the true effect would likely be much larger and therefore more easily detectable in population cancer incidence trends. However, data from the Nordic countries for 1974–2003 (Deltour et al. 2009) and children in the Nordic countries for 1985–2006 (Schmidt et al. 2011) and from Switzerland for 1969–2002 (Roosli et al. 2007), England for 1998–2007 (de Vocht et al. 2011), and the United States for 1992–2006 (Inskip et al. 2010) and for 1987–2007 (Kohler et al. 2011) showed no indication of increases in brain tumor incidence in relation to the introduction and growing use of mobile phones, up to 20 years after their introduction and 10 years after their use became widespread.

This does not appear compatible with the greatest risk shown in the Interphone study—the ORs of about 4 within 5 years of first use for individuals using a phone for ≥1,640 hr cumulatively—or with the risk estimates using a “low user” baseline group, given in the appendix of the Interphone report (Interphone Study Group 2010).

The Interphone levels of exposure were those in the population in 2003 and earlier; since then, prevalence and probably levels of use have increased greatly. Future examination of cancer incidence trend data over the next few years, especially by age of occurrence and anatomic location of tumors, should greatly clarify whether mobile phones cause brain tumors: If there are no apparent effects on trends in the next few years, after almost universal exposure to mobile phones in Western countries, it will become increasingly implausible that there is a material causal effect. Conversely, if there are unexplained rising trends, this will not in itself prove causation, but will give a case to be answered. Supplemental Material, Figure 1 (http://dx.doi.org/10.1289/ehp.1103693), shows the most recently available data, up to 2009, from Sweden, one of the earliest adopters of mobile phones (see Supplemental Material, Figure 2); the data give evidence against an impact of mobile phone use on brain tumor occurrence.

## Conclusions

Interphone is an impressively large study with multiple indices of exposure. However, it has some methodological deficits, largely inevitable in recall-based case–control studies, which limit interpretation of its findings. Such evidence as it provides, combined with the results of biological and animal studies, other epidemiologic studies, and brain tumor incidence trends, suggest that within the first 10–15 years after first mobile phone use there is unlikely to be a material increase in risk of adult brain tumors resulting from mobile phone use. At present there are no data on risk of childhood tumors.

The deficiencies of exposure measurement, because of recall misclassification in studies such as Interphone, and because of misidentification of users in records-based studies such as the published cohorts, leave it doubtful that either study type could reliably detect a small effect, if one existed. Both for this reason and because research cannot in principle prove the complete absence of an effect but only place limits on its possible magnitude, there is bound to remain some uncertainty for many years to come. The limited duration of data yet available, which is mainly for up to 10 years of exposure and to a lesser extent for a few years beyond this, also leave uncertainty because of the potential for long lag period effects, especially for meningioma, which is generally slower growing than glioma. The possibility of a small or a longer-term effect thus cannot be ruled out. Nevertheless, although one cannot be certain, the trend in the accumulating evidence is increasingly against the hypothesis that mobile phone use causes brain tumors.

## Supplemental Material

(104 KB) PDF
