# Supplementary material for: Mobile Phones, Brain Tumors, and the Interphone Study: Where Are We Now?
Source: Environ Health Perspect. 2011 Jul 1;119(11):1534–8. doi: 10.1289/ehp.1103693 (PMC3226506; doi:10.1289/ehp.1103693)
Supplement: (104 KB) PDF [file ehp.1103693.s001.pdf]

Supplemental Material

Mobile Phones, Brain Tumours and the Interphone Study: Where Are We Now?

Anthony J. Swerdlow, Maria Feychting, Adele C Green, Leeka Kheifets, David A Savitz (International Commission for Non-Ionizing Radiation Protection Standing Committee on Epidemiology)

Table of Contents

Factors potentially contributing to diminished ORs in mobile phone users …..….. 1
Published results on cumulative call time from Interphone component studies …. 1
Analogue/digital/cordless phones ……………………………………………… 2
Figure 1: Incidence of glioma, Sweden 1970-2009, (a) males, (b) females ……….3
Figure 2: Mobile phone subscriptions per 100 inhabitants, Sweden, 1987-2004 … 4
References ………………………………………………………………………… 5

Factors potentially contributing to diminished ORs in mobile phone users

As well as non-response bias and prodromal symptoms, reduced ORs in mobile phone users might be due to the following:

Timing of interviews differing between cases and controls combined with strong secular trends in mobile phone use, though this was examined directly in Interphone and found not to contribute; differential misclassification of mobile phone use, but if anything one would expect cases to overreport relative to controls, creating bias toward raised, not diminished, risk for phone users; mobile phone use serving as a marker of socioeconomic or other factors associated with low risk of brain tumour or of its diagnosis. However, the results were adjusted for socioeconomic status, the evidence does not suggest that brain tumours are more common in low social classes, and no other aetiological factor with such an effect is known.

Published results on cumulative call time from Interphone component studies

In the seven individual Interphone component studies (Christensen et al. 2005; Hepworth et al. 2006; Hours et al. 2007; Klaeboe et al. 2007; Lonn et al. 2005; Schuz et al. 2006; Takebayashi et al. 2008) and one combined study (Lahkola et al. 2007) published, there were no statistically significant positive associations with cumulative call time observed and no suggestion of any dose-response gradients.

Analogue/digital/cordless phones

Average output powers from analogue phones have generally been higher than from the digital phones that have replaced them, as analogue phones did not have adaptive power control and because of other technological advances in efficiency. Another difference is that digital phones use pulsed signals. For these reasons, Interphone analysed results for analogue and digital phones separately; however, no consistent differences were found between results for use of these phone types. Similarly, none of the national Interphone publications that published results for analogue and digital phones separately (Hepworth et al. 2006; Klaeboe et al. 2007; Lonn et al. 2005; Takebayashi et al. 2008) indicated any differences in results between analogue and digital phones, despite potential differences in RF exposure from the different phone types. There were greater risks found for analogue than digital use in Hardell et al's data (Hardell et al. 2006a; Hardell et al. 2006b) and, with wide confidence intervals, Auvinen's (Auvinen et al. 2002).

Cordless phones were not included in the analyses of the main Interphone paper, because average output power levels from cordless phones are considerably lower than average output levels from mobile phones. Two of the national Interphone papers did, however, include cordless phone use (Lonn et al. 2005; Schuz et al. 2006), and neither found any indication that such use was related to glioma or meningioma risk. (The results of Hardell et al (2009) were again an outlier, with greatly raised risks). Thus, it seems unlikely that the omission of cordless phone use could have affected the results in the main Interphone paper.

(a) Males

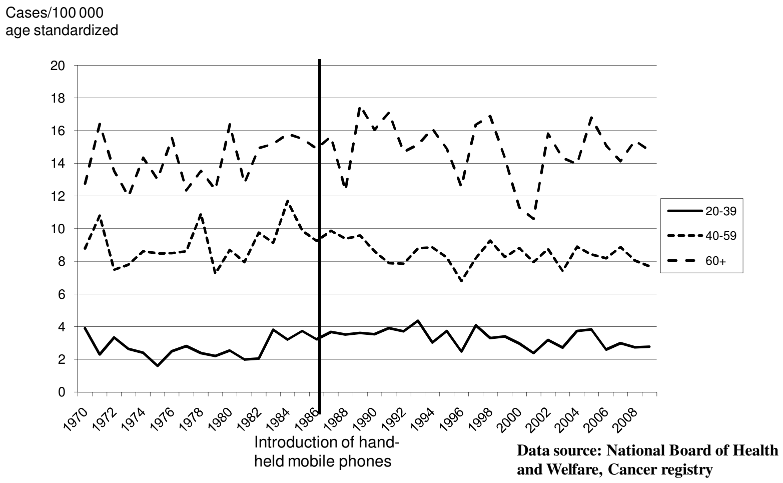

(b) Females

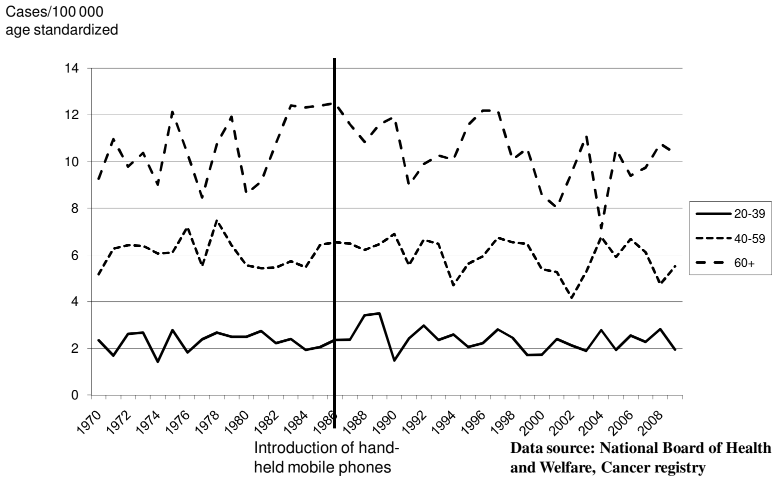

Supplemental Material, Figure 1: Incidence of glioma[a], Sweden 1970-2009, (a) males, (b) females

[a]Based on Swedish cancer registry coding that excludes ependymoma.

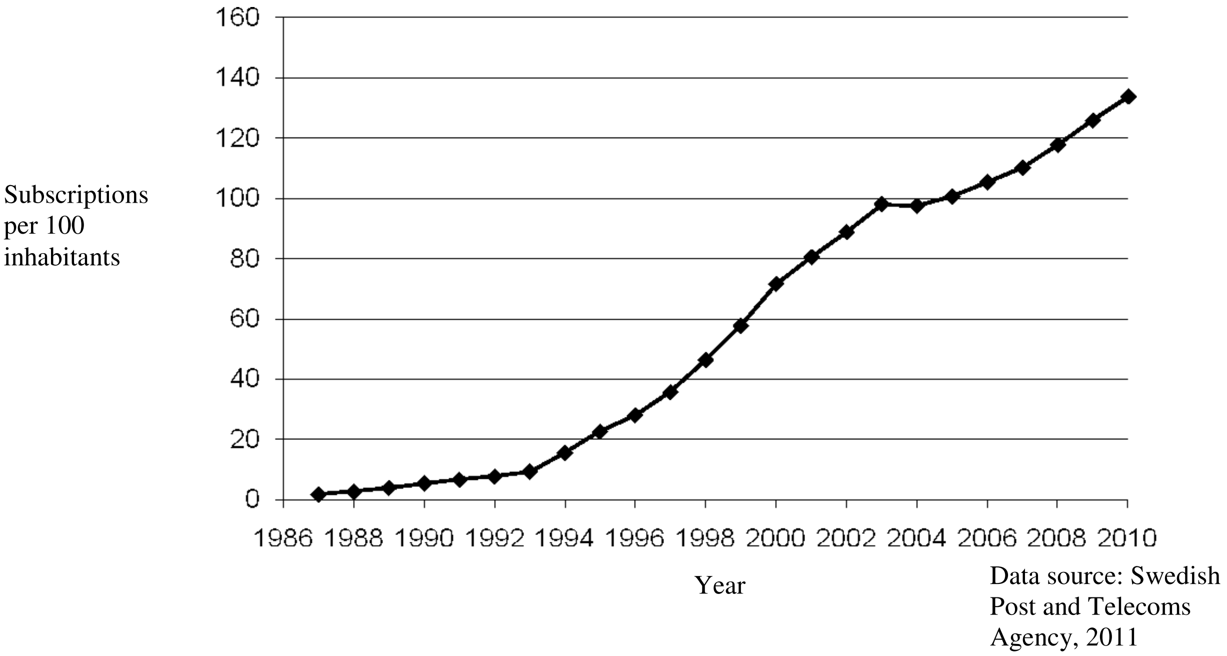

Supplemental Material, Figure 2: Mobile phone subscriptions per 100 inhabitants, Sweden, 1987-2010*

*The disjunction in the trend in 2004 is caused by a change in the definition of what constitutes an "active" pay-as-you-go card

REFERENCES

Auvinen A, Hietanen M, Luukkonen R, Koskela R-S 2002 Brain tumors and salivary gland cancers among cellular telephone users. Epidemiology 13: 356-359

Christensen HC, Schuz J, Kosteljanetz M, Poulsen HS, Boice JD, Jr., McLaughlin JK, Johansen C 2005 Cellular telephones and risk for brain tumors: a population-based, incident case-control study. Neurology. 64: 1189-1195

Hardell L, Carlberg M, Hansson MK 2006a Pooled analysis of two case-control studies on the use of cellular and cordless telephones and the risk of benign brain tumours diagnosed during 1997-2003. Int J Oncol. 28: 509-518

Hardell L, Carlberg M, Hansson MK 2006b Pooled analysis of two case-control studies on use of cellular and cordless telephones and the risk for malignant brain tumours diagnosed in 1997-2003. Int Arch Occup Environ Health. 79: 630-639

Hepworth SJ, Schoemaker MJ, Muir K, Swerdlow AJ, van Tongeren M, McKinney PA, 2006. Mobile phone use and risk of glioma in adults: case-control study. BMJ 332:883-887.

Hours M, Bernard M, Montestrucq L, Arslan M, Bergeret A, Deltour I, Cardis E, 2007. Cell Phones and Risk of brain and acoustic nerve tumours: the French INTERPHONE case-control study. Rev. Epidemiol Sante Publique 55:321-332.

Klaeboe L, Blaasaas KG, Tynes T. 2007. Use of mobile phones in Norway and risk of intracranial tumours. Eur. J. Cancer Prev. 16:158-164.

Lahkola A, Auvinen A, Raitanen J, Schoemaker MJ, Christensen HC, Feychting M, et al. 2007. Mobile phone use and risk of glioma in 5 North European countries. Int. J. Cancer 120:1769-1775.

Lonn S, Ahlbom A, Hall P, Feychting M. 2005. Long-term mobile phone use and brain tumor risk. Am. J. Epidemiol. 161:526-535.

Schuz J, Bohler E, Berg G, Schlehofer B, Hettinger I, Schlaefer K, et al. 2006. Cellular phones, cordless phones, and the risks of glioma and meningioma (Interphone Study Group, Germany). Am. J. Epidemiol. 163:512-520.

Swedish National Board of Health and Welfare 2011. http://192.137.163.40/epcfs/index.asp?kod=engelska [accessed 17 May 2011].

Swedish Post and Telecoms Agency, Statistics Portal. http://www.statistik.pts.se/pts2010/index.html [accessed 24 May 2011].

Takebayashi T, Varsier N, Kikuchi Y, Wake K, Taki M, Watanabe S, Akiba S, Yamaguchi N 2008 Mobile phone use, exposure to radiofrequency electromagnetic field, and brain tumour: a case-control study. Br J Cancer. 98: 652-659
